# A quality improvement initiative using peer audit and feedback to improve compliance

**DOI:** 10.1093/intqhc/mzac058

**Published:** 2022-06-30

**Authors:** Annemarie Fridrich, Anita Imhof, Sven Staender, Mirko Brenni, David Schwappach

**Affiliations:** Swiss Patient Safety Foundation, Asystrassse 77, Zurich 8032, Switzerland; Swiss Patient Safety Foundation, Asystrassse 77, Zurich 8032, Switzerland; Department of Anaesthesia & Intensive Care Medicine, Regional Hospital Maennedorf, Asylstrasse 10, Maennedorf 8708, Switzerland; Institute of Anesthesiology, Intensive Care Medicine, Emergency and Rescue Medicine, See-Spital, Asylstrasse 19, Horgen 8810, Switzerland; Swiss Patient Safety Foundation, Asystrassse 77, Zurich 8032, Switzerland; Institute of Social and Preventive Medicine (ISPM), University of Bern, Mittelstrasse 43, Bern 3012, Switzerland

**Keywords:** checklist, surgery, compliance, observation, feedback, patient safety

## Abstract

**Background:**

The Surgical Safety Checklist (SSC) published by the WHO in 2009 is used as standard in surgery worldwide to reduce perioperative patient mortality. However, compliance with the SSC and quality of its application are often not satisfactory. Internal audits and feedbacks seem promising for improving SSC application.

**Objective:**

The purpose of this study is to investigate whether an intervention consisting of peer observation and immediate peer feedback can be implemented with high fidelity and acceptance.

**Method:**

Data were obtained from a national pilot programme that was initiated in Switzerland in 2018 to measure and improve compliance with the SSC using peer audit and feedback. A total of 11 hospitals with 14 sites implemented the full intervention. Each hospital formed an interprofessional project team that should perform at least 30 observations with feedback on SSC application documented in an observation tool developed specifically for this programme. Since the SSCs of the study hospitals differ greatly regarding checklist items, for each of the three SSC sections standard items were defined: four at Sign In, five at Team Time Out and two at Sign Out. Frequency analyses were performed for initiation characteristics, SSC application at item level, feedback characteristics and programme evaluation.

**Results:**

The 11 hospitals documented 715 valid observations, and feedback on SSC application was provided for 79% of the observations. In 61%, all team members stopped their work for the SSC application, and in 71%, the items were read off from the checklist (instead of recalled from memory). On average, 86% of the standard items were read out by the checklist coordinator, whilst the two items at Sign Out were read out only in 60% and 74%. Additional visual checks with another source (e.g. patient wristband) took place in only 41%, and verbal confirmation of the items (by someone else other than the checklist coordinator) was obtained on an average of 76% across all three checklist sections. The surgical teams reacted positively in 64% to the peer feedback.

**Conclusion:**

Both implementation fidelity and acceptability of the intervention were high—the present intervention seems suitable for regular monitoring of the quality of SSC application with internal resources. Peer observation facilitated identifying weaknesses regarding the SSC process and application at item level. Across all hospitals, the Sign Out section in general, visual control for item checks and lack of work interruption of all team members during SSC application showed up as the main areas of improvement.

## Introduction

The surgical safety checklist (SSC) published by the World Health Organization (WHO) in 2009 [1] is used as standard in surgery worldwide to reduce perioperative patient mortality and complications, but its structure and content [2] as well as quality and consistency of its application vary greatly [3–7]. Routine, time pressure, untrained staff and new procedures can endanger compliance with the SSC and quality of its application. However, it has been shown that positive effects of the SSC require a high level of compliance (i.e. frequency) [3, 4, 8], and the quality of checklist application seems to moderate the SSC’s effectiveness [9]. It is therefore important to monitor SSC application as part of a continuous improvement process.

It has been shown that audits and feedback can improve SSC application [10, 11], but there are many different ways auditing and feedback can be implemented. External audits have the advantage that they are rather objective and that experienced auditors can be involved. However, external auditors are less familiar with internal processes, so they may have greater difficulty following a hospital-specific SSC, for example. In addition, external audits usually only take place at certain points after prior informing of the surgical team, which can possibly lead to increased attention and thus confounding the results [12]. Internal audits, on the other hand, often have a positive bias [12, 13] and require training of internal observers, but internal observers often enjoy better acceptance [14].

Feedback can also be provided in very different ways. Comprehensive feedback in the sense of a debriefing takes time and can therefore not be given immediately after SSC application. Brief feedback directly after SSC application often only focuses on a single element or situation. However, the observed behaviour is still very present, all surgical team members can participate and, if necessary, brief feedback can be supplemented by a debriefing at a later point in time. Empirical findings have shown that acceptance is higher for timely feedback than deferred debriefing and that brief specific feedback is more effective than general and broad feedback [14].

Internal audits and brief feedbacks therefore seem promising for improving SSC application. The purpose of this study is to investigate whether an intervention consisting of peer observation and immediate peer feedback can be implemented and how this feedback is accepted by the teams observed. Regarding the intervention success, we focus on two implementation indicators [15]: first, fidelity is studied, i.e. whether the intervention could be implemented as planned. Second, acceptance of both, the observing and the observed teams are studied. Furthermore, the results from observation and feedback on the quality of SSC application will be reported.

## Methods

**Figure 1:**
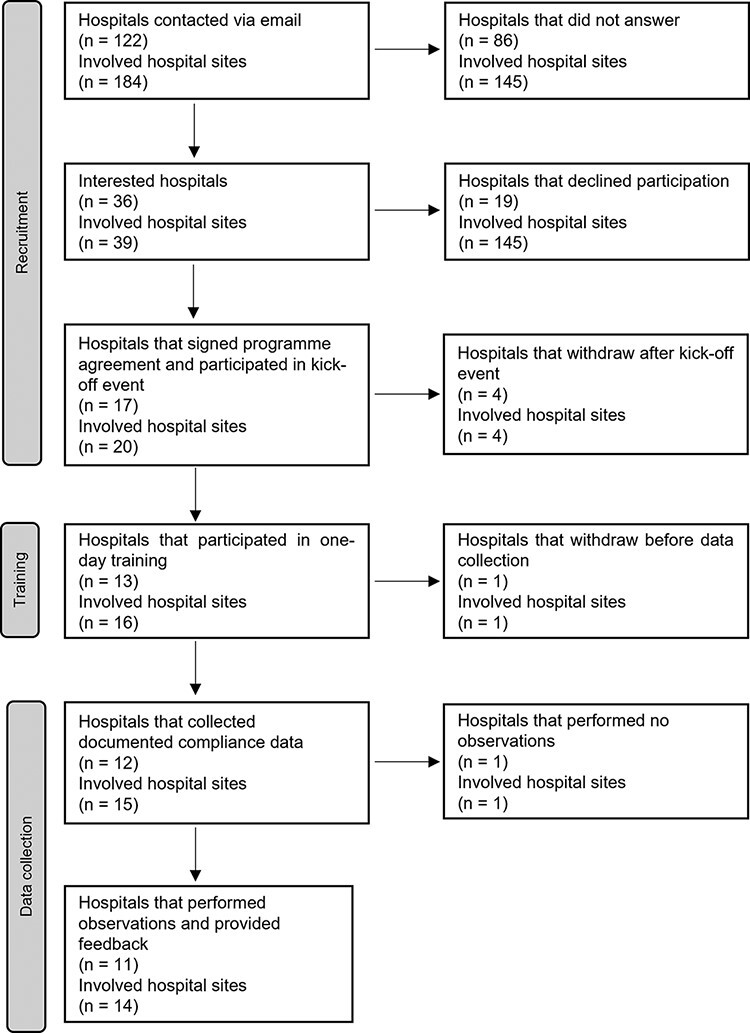
Sample size during recruitment, training and data collection phase.

Data for this study come from a national pilot programme that was initiated in Switzerland in 2018 to measure and improve compliance with the SSC. Hospital participation was voluntary. A total of 11 hospitals with 14 sites implemented the full intervention. See Figure 1 for more detailed information on the sample size during recruitment, training and data collection.

Each hospital formed an interprofessional project team, consisting of at least an anaesthesiologist, a nurse and a surgeon. All teams received a 1-day training on observation and feedback. Each team was expected to perform 30 observations on SSC application and provide immediate feedback to the surgical team. Thus, each team designated multiple observers, but each observation was made by only one person at a time. Observations should be evenly distributed among the three professional groups (surgeons, anaesthetists and nurses) and the three SSC sections, Sign In (applied before induction of anaesthesia), Team Time Out (applied before skin incision) and Sign Out (applied after surgery).

### Observation tool

An observation tool was developed, which included standard items and criteria, but was easily adaptable to local needs and specific SSCs. It was based on existing tools such as the Checklist Usability Tool [5], the WHOBARS [16], findings from a national study on structured intraoperative briefings [17], elements of Closed Loop Communication [18, 19] and basics of the anatomy of the SSC [20]. The observation tool was tested in two hospitals: one person from the programme team and one person from the respective hospital (surgeon, anaesthetist or nurse) simultaneously observed the SSC application. The mean interrater reliability was *к *= 0.63. It was found that at least three trial runs in pairs should take place before the actual observations. Furthermore, the pretest demonstrated that it does not seem advisable to conduct more than five observations in a row, as fatigue can occur afterwards.

The tool could be completed electronically or on paper (and digitalized afterwards). It was divided into five parts: general indicators, initiation process, verification of SSC items, overall appraisal of SSC application and feedback.

### General indicators

Data on general indicators had to be completed immediately before the observation. They included profession of the observer, date, time (night: after 4.59 PM or before 7.30 AM [21]), surgical discipline, type of anaesthesia and planning of surgical intervention (elective/emergent).

### Initiation process

Checklist initiation comprised seven criteria: clear initiation (yes/partially/no), checklist lead (profession), correct time of initiation (yes/too early/too late), all present (yes/no), missing team members (profession), all stop work (yes/partially/no) and items read off from checklist instead of recalled from memory (yes/partially/no).

### Verification of SSC items

Observers had to document each item if it was read out by a predefined person (so-called checklist coordinator), visually checked with another source (e.g. patient wristband) and verbally confirmed (by someone else other than the checklist coordinator). The requirements for a correct visual check or a sufficient response were not defined in the observation tool but determined by the guidelines of the respective hospital.

### Overall appraisal of SSC application

Five criteria for the overall appraisal of the SSC application were each assessed on a scale from 1 to 5: checklist lead (1 ‘no lead’; 5 ‘clear lead’), team engagement (1 ‘passive, uninvolved’; 5 ‘active, involved), atmosphere (1 ‘tense, irritated ‘; 5 ‘open, appreciative’), rhythm (1 ‘rushed’; 5 ‘calm’) and acoustic comprehensibility (1 ‘not understandable’; 5 ‘well understandable’).

### Feedback

The feedback part comprised seven criteria: feedback given (yes/postponed/no), reasons for no feedback (acute situation/time pressure/tension in the team/feedback refused/other), feedback topic (topic), feedback focus (positive reinforcement/potential for improvement/ambiguities), feedback follow-up needed (yes/no), reaction to feedback (mainly positive/neutral/mixed/mainly negative) and feedback duration (<1 min/1 to 3 min/>3 min).

### Peer feedback concept

The feedback had to be feasible in everyday surgical practice. Based on expert interviews, the following requirements emerged: The feedback should be given immediately after the observation to the entire team, and the feedback should not last longer than 3 min. The applied feedback concept was based on the three-step technique from the Safe Surgery Checklist Implementation Guide [20]:

*Observation:* First, it should be described what was specifically observed. The description should be as specific, clear and objective as possible.

*Opinion:* Second, the feedback giver formulates their personal view on the observation in a transparent way. This can be in the form of an appraisal, a statement or a transfer of knowledge.

*Question:* Third, with an open and non-judgemental question, the participants are given the opportunity to actively present their own view and draw conclusions.

### Training

All observers (*n* = 74) attended a one-day training where they learned how to use the observation tool and practised giving feedback using the three-step technique. The trainings were led by a member of the programme team and an external feedback expert.

### Data collection

Originally, the data collection was planned for 3 months. Due to COVID-19 and the associated restrictions on surgeries, data collection was extended by 2 months, finally lasting from November 2020 to March 2021.

### Programme evaluation

In September 2021, the members of the project teams were invited by email to evaluate the programme (*n* = 74; 3 persons had left the hospitals). The evaluation comprised 27 statements (e.g. ‘the implemented measures contribute to quality improvement’) that should be rated on a 5-point Likert scale from 1 ‘does not apply at all’ to 5 ‘applies completely’.

### Data analysis

Stata/BE 17.0 was used to perform descriptive analyses. Observations were excluded if no single SSC item was assessed, as here the reliability of the observation was in question. Frequency analyses were performed for initiation characteristics, SSC application at item level and feedback characteristics, overall and separately for each SSC section. Since the SSCs of the study hospitals differ greatly regarding checklist items, for each checklist section standard items were defined that should be included in any SSC: four at Sign In, five at Team Time Out and two at Sign Out (Table 2). For these items, frequency analyses regarding the completeness of item verification (read out/visually checked/verbally confirmed) were performed. Means and standard deviations were calculated for the overall appraisal of the SSC application. Frequency analyses were also performed for the data from the programme evaluation.

## Results

### General characteristics of the sample

The hospitals documented 731 observations, of which 16 (2.2%) were excluded because no SSC item was evaluated. The remaining 715 observations were distributed rather equally among Sign In (*n* = 210), Team Time Out (*n* = 281) and Sign Out (*n* = 224). Nurses took over most of the observations (*n* = 255), followed by operating theatre management/technical staff (*n* = 199), anaesthetists (*n* = 167) and surgeons (*n* = 72). Most observations were conducted in German-speaking (41%) and Italian-speaking (39%) hospitals, with 20% in French-speaking hospitals. The observations were conducted mostly during the day (94%), on weekdays (96%) and at elective procedures (86%). The majority of surgeries observed were on adults (84%) and under general anaesthesia (70%). A wide variety of surgical disciplines were observed, ranging from general/visceral surgery (26%), orthopaedics/traumatology (25%), gynaecology/obstetrics (16%) to ophthalmology (11%) and other disciplines (29%). In 12 observations, the checklist was not applied, which corresponds to a compliance of 98% (*n* = 703).

### Initiation characteristics

Overall, the initiation of the checklist was announced clearly in most cases (85%) and was done at the correct time (90%), with small differences between the SSC sections (Table 1). Whilst in most cases all relevant team members were present (88%), they interrupted their work for the SSC application in only 61% of the cases; observations from the Sign Out showed that even here, only in 50% of the cases everyone stopped their work. In 71% of all observations, the items were read off (instead of recalled from memory), but only in 61% at Sign In. Across all three SSC sections, the checklist was most frequently led by the circulating nurses (30%), followed by the surgeons (25%) and the nurse anaesthetists (24%). Whilst the Sign In was most often led by nurse anaesthetists (64%), the circulating nurses most often led the Team Time Out (51%), and the surgeons most often led the Sign Out (43%). See Table 1 for further details on checklist initiation.

**Table 1:** Observed initiation characteristics

Initiation characteristic	Observations (%) at Sign In (*n* = 210)	Observations (%) at Team Time Out (*n* = 280)	Observations (%) at Sign Out (*n* = 213)	Total observations (%) (*n* = 703)
Clear initiation				
Yes	162 (77%)	256 (91%)	178 (84%)	596 (85%)
Partially	45 (22%)	22 (8%)	28 (13%)	95 (14%)
No	3 (1%)	2 (1%)	7 (3%)	12 (2%)
Correct time of initiation				
Yes	185 (88%)	244 (87%)	206 (97%)	635 (90%)
Too early	18 (9%)	33 (12%)	3 (1%)	54 (7%)
Too late	7 (3%)	3 (1%)	4 (2%)	14 (2%)
Checklist lead				
Surgeon	0 (0%)	82 (29%)	92 (43%)	174 (25%)
Anaesthesist	60 (29%)	3 (1%)	3 (1%)	66 (9%)
Nurse anaesthetist	135 (64%)	23 (8%)	13 (6%)	171 (24%)
Circulating nurse	3 (1%)	144 (51%)	63 (30%)	210 (30%)
Other	4 (2%)	2 (1%)	20 (9%)	26 (4%)
Multiple persons	8 (1%)	26 (9%)	22 (10%)	56 (8%)
All present				
Yes	172 (82%)	256 (91%)	188 (88%)	612 (88%)
No	38 (18%)	24 (9%)	25 (12%)	87 (12%)
All stop work				
Yes	135 (64%)	191 (68%)	106 (50%)	432 (61%)
Partially	65 (31%)	77 (28%)	76 (36%)	218 (31%)
No	10 (5%)	12 (4%)	31 (15%)	53 (8%)
Items read off from checklist (instead of recalled from memory)				
Yes	128 (61%)	209 (75%)	162 (76%)	499 (71%)
Partially	48 (23%)	40 (14%)	24 (11%)	112 (16%)
No	34 (16%)	31 (11%)	27 (13%)	92 (13%)

### SSC application at item level

Table 2 shows how often the standard items were read out, visually checked and verbally confirmed. Whilst 8 of the 11 standard items were each read out in over 92% of the cases, the Sign In item ‘Allergies’ was read out in only 72% of the cases and the Sign Out standard items only in 60% (name of the procedure) and 74% (postoperative care), respectively. The item ‘Identity of the patient’ was read out in almost all observations at both Sign In (98%) and Team Time Out (97%) but visually checked in only 76% and 45%, respectively. This is similar to the other items: all items were visually checked less frequently (on average 41%) than read out (on average 86%), whilst the proportion of visual checks ranged from 17% (Sign Out: name of the procedure) to 76% (Sign In: identity). On average, the 11 standard items were verbally confirmed in 76% of the observations ranging from 42% (Sign Out: name of the procedure) to 92% (identity).

**Table 2:** Results from the observations on the verification of the standard items

Checklist section	Item name	Read out^a^	Visually checked^b^	Verbally confirmed^c^	Sample size
Sign In	Identity	98%	76%	92%	199
	Procedure	92%	63%	86%	189
	Site mark	94%	62%	86%	200
	Allergies	72%	61%	64%	170
Team Time Out	Identity	97%	45%	83%	280
	Procedure	98%	31%	84%	280
	Site (mark)	84%	32%	71%	280
	Risks surgeon	88%	24%	79%	270
	Risks anaesthesia	87%	17%	76%	255
Sign Out	Name of the procedure	60%	17%	42%	213
	Postoperative care	74%	20%	69%	204

a By the checklist coordinator.

b With another source, e.g. patient wristband.

c By someone else than the checklist coordinator.

### Overall appraisal of the SSC application

**Figure 2:**
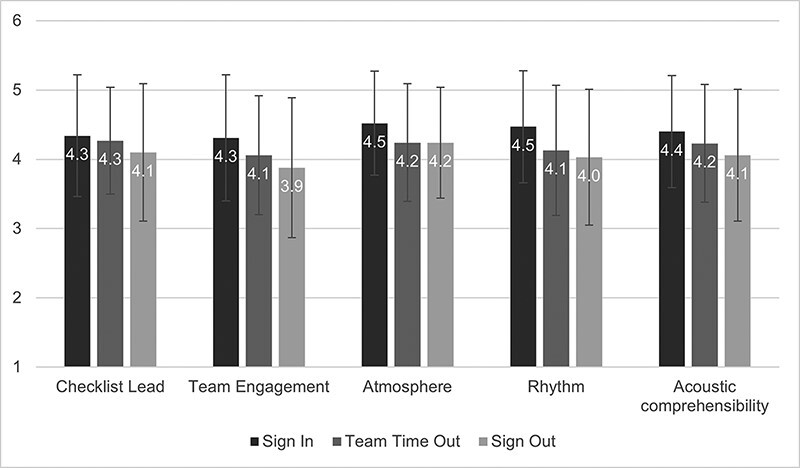
Overall appraisal of the checklist application regarding five characteristics for each checklist section (Sign In: *n* = 210; Team Time Out: *n* = 280; Sign Out: *n* = 213). Vertical error bars indicate the standard deviation.

The SSC application was rated most positively in terms of atmosphere (*M*, 4.32; SD, 0.81) followed by checklist lead (*M*, 4.23; SD, 0.88), acoustic comprehensibility (*M*, 4.23; SD, 0.88), rhythm (*M*, 4.20; SD, 0.93) and team engagement (*M*, 4.08; SD, 0.93). Figure 2 shows the overall appraisal of the SSC application for each checklist section.

### Feedback characteristics

Feedback was given in 79% (*n* = 565) of all observations. Time pressure (52%) was documented as the main reason why feedback could not be provided; feedback was explicitly rejected in only 25 of the 565 observations (3%). The feedback lasted 1–3 min in 96% of all cases, and in most cases (85%), no follow-up was needed. Half of the feedback (51%) focused on reinforcing positive behaviour, 31% on potential for improvement and 18% on discussing ambiguities. The reaction of the surgical team to the feedback as perceived by observers was mainly positive in 61% of the cases, mixed or neutral in 35% and mainly negative in only 1%. Table 3 provides details on feedback for the three SSC sections.

**Table 3:** Documented feedback characteristics

Feedback characteristic	Number (%) for Sign In	Number (%) for Team Time Out	Number (%) for Sign Out	Total number (%)
Feedback given				
Yes	154 (73%)	228 (81%)	183 (82%)	565 (79%)
Postponed	7 (3%)	2 (1%)	5 (2%)	14 (2%)
No	49 (23%)	51 (18%)	36 (16%)	136 (19%)
Reasons for no feedback				
Time pressure	30 (54%)	33 (62%)	15 (37%)	78 (52%)
Feedback refused	1 (2%)	11 (21%)	13 (32%)	25 (17%)
Other	25 (45%)	9 (17%)	13 (32%)	47 (31%)
Feedback duration				
<1 min	88 (57%)	169 (74%)	125 (68%)	382 (68%)
1–3 min	55 (36%)	58 (25%)	48 (26%)	161 (29%)
>3 min	11 (7%)	1 (0%)	10 (5%)	22 (4%)
Feedback follow-up				
Yes	23 (15%)	26 (11%)	36 (20%)	85 (15%)
No	131 (85%)	202 (89%)	147 (80%)	480 (85%)
Feedback focus				
Positive reinforcement	79 (51%)	112 (49%)	98 (54%)	289 (51%)
Potential for improvement	41 (22%)	74 (32%)	60 (33%)	175 (31%)
Ambiguities	34 (27%)	42 (18%)	25 (14%)	101 (18%)
Reaction to feedback				
Mainly positive	113 (73%)	147 (64%)	101 (55%)	361 (64%)
Neutral/mixed	40 (26%)	81 (36%)	79 (43%)	200 (35%)
Mainly negative	1 (1%)	0 (0%)	3 (2%)	4 (1%)

### Programme evaluation

In total, 27 (38%) of the 71 programme participants completed the evaluation questionnaire. Ninety-three percent agreed that participation in the programme was worthwhile and that the effort required to perform 10 observations with immediate feedback per person was feasible. Adapting the programme’s measures to the conditions and needs of the respective hospital was important or very important for 96%. Seventy-eight percent said they felt comfortable observing and giving feedback. The majority of the participants stated that they would continue the peer observations (63%), the immediate feedback (52%) and the interprofessional project group (81%) in their organization.

## Discussion

### Statement of principal findings

The aim of this study was to investigate whether a peer observation and feedback intervention on the application of the SSC can be implemented and how this feedback is accepted by the teams observed. Overall, the implementation fidelity was high. Eleven of the 12 hospitals performed observations and provided feedback; only one hospital did not implement the intervention. On average, these 11 hospitals documented 65 observations; all met the minimum of 30 observations. In 79% of the observations, feedback could be given. Anaesthetists, nurses and surgeons should each perform one-third of the observations; only observers from surgery did not meet this requirement.

The acceptance of the intervention was good. The feedback was explicitly rejected only rarely and the surgical team mostly reacted positively to the peer feedback. The programme evaluation showed that almost all responding participants considered the programme worthwhile and that the effort required for the implementation was feasible. There was also a positive tendency regarding sustainability as the majority would like to continue the programme measures.

About the initiation process, it is noticeable that often not all team members interrupted their work for the SSC application, and items were often not read off from the checklist but recalled from memory.

The item checks showed that the standard items at Sign In and Team Time Out are often read out by the checklist coordinator, but visual checks with another source are often neglected. Especially the Sign Out standard items are often not read out and even less often visually checked and verbally confirmed by another person than the checklist coordinator.

### Strengths and limitations

The present study is an observational study based on self-reported data. Therefore, no statements can be made about the objectivity and reliability of the data, but the results of the pretest indicate adequate psychometric properties of the instrument. Due to the voluntariness of participation, there might have been a self-selection bias.

The focus of the intervention was on building internal competences and the impetus for quality improvement and not on the evaluation of effectiveness. As a result, many improvements could already be implemented before the actual data collection, and the participants’ motivation to change was very high during the entire programme, as every idea for improvement could be implemented immediately.

There was little variance in the overall appraisal of SSC application. It is possible that the observers found it difficult to rate the checklist application by their own colleagues on a continuum from good to bad. Here, the answer format should be reconsidered.

The response rate to the programme evaluation was rather low, so the informative value must be assessed with caution.

### Interpretation within the context of wider literature

Some findings are in line with previous studies. For example, other studies [5, 6, 22] have already shown that the Sign Out is the section with the most potential for improvement. An observational study by Russ et al. [5] found that in 70% of the cases, not all team members interrupted their work for SSC application, which is even higher than the results of the present study showed (39%).

Differences were found, for example, in the quality of item checks. While Cullati et al. [22] found that verbal validation was often missing, in the present study, verbal confirmation by another person other than the checklist coordinator was observed frequently, but visual checks with another source were missing for many of the standard items. The compliance rate of 98% is significantly higher than average compliance (75%) reported in a review [3]. Other studies have already shown that data from self-assessments are higher than data from independent audits [13]; thus, it does not seem unlikely that compliance was overestimated in the present study.

### Implications for policy, practice and research

Bringing interprofessional teams together and facilitating exchange among them emerged as a success factor. But even though all hospitals set up interprofessional teams, surgeons have been rather underrepresented in the main parts of the project. Therefore, the question of how they can be better involved in future quality improvement projects remains open.

Based on the results of the programme evaluation and the exchange of experiences with the participating hospitals, we assume that our intervention has had positive effects. For example, it was reported that checklists and guidelines were adapted; training on checklist application and regular monitoring was planned. We also expect that involving team members in checklist evaluation and feedback would contribute positively to cultural change.

Nevertheless, as immediate learning effects at the individual or team level could not be identified within the available data, a study with a before–after design with tracking performance of specific teams or individuals should confirm the effectiveness of the intervention.

Across all hospitals, the Sign Out section in general, visual control for item checks and lack of work interruption of all team members during SSC application showed up as the main areas of improvement. Interventions addressing these weaknesses should be developed in future.

## Conclusions

Peer observation facilitated the identification of weaknesses regarding the SSC process and SSC application at item level. These weaknesses could be communicated directly through peer feedback. Both implementation fidelity and acceptability were high—the present intervention therefore seems suitable for regular monitoring of the quality of SSC application with internal resources. However, peer observation is not recommended for measuring general compliance in terms of ‘SSC applied vs. not applied’; more objective methods should be used for this.

## Data sharing statement

Data are available on reasonable request.
